# Assessing the knowledge and practice toward food safety: An investigation of food selection and processing among primary food caregivers in a town of Ha Tinh province, Vietnam

**DOI:** 10.1016/j.heliyon.2023.e20004

**Published:** 2023-09-09

**Authors:** Ngoc Quang La, Minh Luan Hoang, Thi Tao Tran, Cao Khoa Dang, Binh Thang Tran

**Affiliations:** a Department of Epidemiology, Hanoi University of Public Health, Hanoi, Viet Nam; b Ky Anh Town Health Center, Ha Tinh Province, Viet Nam; c Faculty of Public Health, University of Medicine and Pharmacy, Hue University, Hue city, Viet Nam

**Keywords:** Knowledge, Practice, Food selection and processing, Food caregivers

## Abstract

The food selection and processing stage are important stages to prevent food poisoning. A good level of knowledge and practice regarding food selection and processing among people who are responsible for the family meals are important. In this study, we aimed to investigate the knowledge and practice of primary food caregivers regarding food poisoning prevention in food selection and processing and identify the factors that influence these outcomes. The current study applied a cross-sectional study to investigate 422 primary food caregivers in urban areas in Vietnam. Data were collected using a structured questionnaire, and knowledge and practice were assessed based on pre-defined criteria. The data were analyzed using descriptive statistics, chi-square test, and logistic regression. Our study found that 78.9% of people had good knowledge regarding food poisoning prevention in food selection and processing. Furthermore, 84.4% of people had correct practice in this regard. Our study also revealed that household income level and educational level were associated with knowledge and practice, respectively. The proportion of correct practice was higher in the group with good knowledge (90.4%) compared to the remaining group (61.8%). The results indicated that the proportion of good knowledge and practice were high among food caregivers, but further efforts are needed to improve the knowledge and practice of food caregivers with lower household income and educational levels. The findings also emphasize the importance of communication campaigns to enhance knowledge related to food poisoning prevention.

## 1 Introduction and study background

Food poisoning is an acute illness caused by contaminated foods with bacteria, viruses, parasites or harmful chemicals [1]. Food poisoning is one of the health issues in relation to food safety, which has been drawn much attention from public health in recent years. According to World Health Organization (WHO), foodborne diseases have been estimated to be about 30% of population in high-income countries. Notably, the burden has been recognized to be more severe in developing countries with two million deaths/year [2] and Vietnam is no exception. The situation of food poisoning and foodborne illness is a significant concern in Vietnam. In detail, a total of 170 cases reported yearly with 5000 infected people and 27 deaths due to food poisoning, especially, there were 7 foodborne illnesses reported with 4 million infected people and 123 deaths during the period 2011 to 2016 [3].

There are many causes leading to food poisoning. Food contamination may occur at any stage of the entire process from the farm to the table, including in the kitchens of consumers themselves. Thus, it is necessary to implement interventions to prevent food illness at every step throughout the process of food preparation [4]. It should be noted that 85.6% of food processing takes place in households, which is responsible for around half of reported cases of food poisoning [5]. As a result, food preparation at home is a main source of food poisoning. However, a majority of people believe that food poisoning results from restaurants, food facilities due to unsafety in food processing and selection, whereas there was little or no awareness on the impact of their kitchens on food poisoning [5].

To date, the important role of food handlers in the transmission of foodborne illness has been emphasized due to their knowledge about food safety [6,7]. Few studies have been conducted to investigate knowledge and practice regarding food safety among food caregivers. The existing evidence supports the argument that food handlers have incorrect knowledge and practices regarding food safety at household levels. For example, a study in Egypt presented the poor knowledge in food preparation and incorrect practice in purchasing and storage of women working in an university [8]. Home environment was also recognized to be of great importance for the spread of foodborne diseases [9] with moderate level of awareness among housewives [10]. Thus, poor knowledge and incorrect practices of food handlers in general and food caregivers in particular could associate with foodborne disease transmission.

In comparison with high income countries, foodborne disease burden were reported to be higher in low-and middle-income countries (LMICs) [11]. Notably, foodborne diseases seem to increase in LMICs due to the significant rise in risky food consumption and the expansion of value chains [12]. These diseases are attributed to incorrect or improper food hygiene and handling practices at home, which increase due to a high consumption of fats and animal foods in LMICs [13]. However, the importance of food safety has been neglected in LMICs [12]. Food handlers were indicated to have little knowledge related to this issues [14]. A study in Bangladesh showed the low level of overall knowledge and practices about food safety [14]. Additionally, a total of 33% of households in India did not have a refrigerator and correct practices regarding food storage were observed only in 12.6% of households with a refrigerator [13]. Similarly, a relatively high prevalence of undesirable practice of food safety was exhibited among Iranian household [15]. In Vietnam, food-borne diseases attract attention due to repeated episodes of adulterated and hazardous foods [16]. Street food vendors were found to have a low level of food handling practices in Vietnam [17]. A lack of food hygiene and safety knowledge was reported for three domains, which includes standard requirements for food facilities, food poisoning prevention, and food processing procedures [18]. Notably, many foodborne illness cases are associated with home environments as a result of the improper handling and preparation of food among food caregivers [19]. Additionally, it is important to consider the vulnerable population including elderly, pregnant, and children who reside at home and are more susceptible to the detrimental effects of foodborne illness [19]. However, the majority of previous studies focused on food sellers [17,18]. In contrast, food safety at home, which is considered as the last line of foodborne disease prevention [19], has been neglected in Vietnam. Thus, our study aimed to provide useful insights into the knowledge and practice and to explore the related factors among food caregivers in food selection and processing at household level in central region in Vietnam.

## 2 Study method

### 2.1 Study subjects

A household food caregivers is defined as a person who was responsible for daily meals and groceries of the family.

### 2.2 Setting and time for data collection

The study was carried out in Hung Tri ward, which is located in Ky Anh town, Ha Tinh province, from January to September 2022. Ky Anh town is a part of Ha Tinh Province, a province in the North Central Coast region of Vietnam. Hung Tri ward is situated at the center of Ky Anh Town and is considered one of the most dynamic economic zones of the town, with a population of approximately 15,447 people [20].

### 2.3 Study design

The study used a cross-sectional design.

#### 2.3.1 Sample size and sampling method

Using the formula to estimate the minimum sample size of a proportion in the population, the calculation is:

$$n=Z_{1-\alpha /2}^{2}\;X\;\frac{p\;\left(1-p\right)}{d^{2}}$$

n: Minimum sample size of the study

Z:Distribution factor at (1-α/2) confidence level

p:Estimated proportion of the population

d:Precision level

With estimated proportion for this formula, we adapted the finding from a similar study in Duc Thanh commune, Duc Tho district, Ha Tinh province in 2018 (47.4% of housewives had good knowledge about food safety). To achieve a desired error of 5% (0.05), a sample size of 383 was needed. Finally, a total sample size of 422 food caregivers was involved [21].

#### 2.3.2 The sample was selected through a multi-stage sampling method

**Phase 1:** Selected randomly five out of 11 residential quarters in Hung Tri ward.

**Phase 2:** Applied probability-proportional-to-size sampling to calculate the size for each residential quarter. We applied the simple random sampling for each selected quarter.

### 2.4 Data collection method

The research team obtained the list of households in the 5 selected residential quarters from local commune health center. All the interviews were performed face-to-face with direct observations using a checklist to assess practices of the food caregivers. All data collectors were trained before performing data collection.

### 2.5 Questionnaire and measurements

The questionnaire includes: General information of food caregivers such as age, gender, occupation, and education levels; knowledge and practice of food selection and processing of the food caregivers (the questionnaire was developed and adapted using the 10 golden principles of the WHO on Food Safety [22]).

#### 2.5.1 Knowledge assessment criteria

The knowledge of food poisoning prevention in the selection and processing of food was assessed through 25 questions. Based on the total score, we divided into ***Good*** and ***Ingood*** knowledge. The maximum score for the 25 questions is 33 points. The food caregivers who answered correctly and scored 80% or more of the total score (≥26 points) was evaluated as “***Good***".

#### 2.5.2 Practice assessment criteria

The practice is evaluated based on the total score of 21 questions related to the practice. Based on the practice score, we divided into ***Correct*** and ***Incorrect*** practice. The maximum score for the practice is 21 points. The participant who scored 80% or more of the total score (17 points) was evaluated as “***Correct***".

### 2.6 Statistical analysis

Data were processed using SPSS 20.0 software. Descriptive statistics was summarized as frequency and percentage. The Chi square (ꭓ^2^) and Fisher's exact test was statistical test for the bivariate analysis of the association of factors to knowledge and practice with a significance level of α = 0.05. The logistic multiple regression model was used to control for confounding variables. The results were presented by the odds ratio and 95% confidence interval (OR, 95% CI) with statistically significant test result as p < 0.05. Independent variables entered into the logistic multiple regression model using step method with p < 0.3 in the univariate analysis.

## 3 Result

### 3.1 Characteristic of the study participants

Table 1 represents characteristic of the study participants**.** The majority of participants were ≥40 years of age, accounting for 69.7%. A higher proportion was found for females compared to males (86.3% vs. 13.7%). Regarding to educational level, college or higher group has a higher proportion than the remaining groups. In addition, we found a low proportion of food caregiver with low household income (6.4%). Small business and government officer were two major occupations in our participants, 33.9% and 27.3%, respectively. Among of food caregivers, 86.7% were found to have more than 10 years experience for this role.

**Table 1:** Characteristic of the study participants (n = 422).

Characteristics		Frequency (n)	Percentage (%)
Age (years)	<40	128	30.3
	≥40	294	69.7
Sex	Males	58	13.7
	Females	364	86.3
Educational level	Elementary school or illiteracy	10	2.4
	Secondary school	66	15.6
	High school	153	36.3
	College or higher	193	45.7
Occupation	Agriculture	59	14.0
	Small business	143	33.9
	Government officer	115	27.3
	Housewife	61	14.5
	Other	44	10.3
Low household income	Yes	27	6.4
	No	395	93.6
Duration of food caregivers	<5 years	14	3.3
	5–10 years	42	10.0
	>10 years	366	86.7

Television was a main channel to provide information on food safety in our population (87.2%), followed by radio (55.2%). Furthermore, 55.2% and 46.9% of food caregivers obtained this information through friends/relatives and newspapers/magazines, respectively. Meanwhile, we observed 1.9% of food caregivers who have never obtained information on food safety.

### 3.2 The knowledge of food poisoning prevention in food selection and processing of food caregivers

Table 2 provides detailed knowledge regarding food poisoning prevention in food selection and processing among food caregivers. The majority of respondents had correct knowledge regarding the signs of fresh vegetables (88.2%), fresh meat (93.4%), and fresh fish (92.2%). Participants also had good knowledge related to various aspects of food hygiene such as soaking and washing vegetables (92.4%), cooking skills (97.6%), and reheating leftovers (98.1%). They also had high awareness about sorting raw and cooked foods, using separate utensils (99.8%), and keeping food preparation areas clean (99.3%). However, food caregivers had poor knowledge related to the potential risks of using rancid food and unclean drinking water (98.6%). Among the 422 participants included in our study, 78.9% demonstrated a good level of knowledge.

**Table 2:** The knowledge of food poisoning prevention in food selection and processing of food caregivers (n = 422).

Characteristics	Frequency (n)	Percentage (%)
Signs of fresh vegetables		
**Vegetables are plump, not crushed**	372	88.2
**Reasonable colors**	306	72.5
**Reasonable sense of smell**	229	54.3
**Others**	6	1.4
**Unfamiliar**	2	0.5
Signs of fresh meat		
**Bright red color**	394	93.4
**Reasonable sense of smell**	290	68.7
**Resilient**	238	56.4
**Dry slash**	106	25.1
**Others**	2	0.5
**Unfamiliar**	3	0.7
Signs of fresh fish		
**Clear eyes**	389	92.2
**Bright red gills**	346	82.0
**Bright red slash**	248	58.8
**Iridescent scales**	163	38.6
**Unfamiliar**	4	0.9
Eating cooked drinking boiled, soaking, and washing raw vegetables and fruits		
**Vegetables should be soaked in clean water and then washed 3–4 times or washed under running water**	390	92.4
**Food should be cooked thoroughly**	412	97.6
**Eat meal immediately after cooking or right after it has been prepared**		
**When *food is cooked,* it *should* be *eaten promptly***	417	98.8
**Leaving cooked food to cool for a long time is susceptible to food poisoning**	406	96.2
**Nutrient-rich dishes such as meat, fish, eggs, etc., after being processed, which can cause food poisoning if not eaten immediately and well preserved**	408	96.7
Covering up and carefully preserving food after cooking		
**Food should be covered to avoid flies, and, cockroaches, rats**	421	99.8
**Leaving fertilizers, pesticides … near food can cause poisoning**	408	96.7
Carefully reheating leftovers before using again		
**It is hygienic to reheat food before eating**	414	98.1
***Food can* be *contaminated* when it is cool**	411	94.8
Separating raw and cooked food, and separating utensils to prepare food		
**Raw foods are often contaminated with disease-causing microbes**	400	94.8
***Cross-contamination occurs* when juices from uncooked foods come in contact with safely cooked foods**	417	98.8
**Use different utensils, plates and chopping boards for raw and cooked food**	419	99.3
**Wash utensils before preparing food**	419	99.3
**Leftover should be stored covered and remove daily**	420	99.5
Washing hands before handling food, keeping utensils and food preparation tables dry		
**Washing hands before handling foods and after using the *restroom***	421	99.8
**Food preparation tables must be dry, far from latrines, chicken, duck and pig coops at least** > **10m**	414	98.1
**Places of eating, processing, pantry with flies, cockroaches and insects are unhygienic**	419	99.3
**Keep food preparation tables away from disinfectants, disinfectants, fertilizers, pesticides**	417	98.8
**Food preparation tables need maintained dry and sanitary**	420	99.5
Using rancid food and clean water		
**Using spoiled food can cause illness**	421	99.8
**Leaving cooked food to cool for a long time is susceptible to food poisoning**	415	98.3
**Lack of clean water for processing food will be unhygienic**	416	98.6
Overall Knowledge		
***Good***	*333*	***78.9***
***Ingood***	*89*	*21.1*

### 3.3 Practice about food poisoning prevention in food selection and processing

Table 3 provides detailed information on the practices related to food poisoning prevention in food selection and processing among food caregivers. A majority of participants reported buying food in the morning (77.5%) and buying enough food for their needs (58.8%). In terms of choosing healthy food, participants were knowledgeable about the signs of fresh produce and seafood, with over 85% correctly identifying signs of freshness. Participants also demonstrated good knowledge about food preparation, including soaking and washing vegetables, cooking food thoroughly, and separating utensils for raw and cooked foods. The majority of participants reported correct practices in covering and preserving cooked food, as well as maintaining sanitary conditions at the food processing table. However, there is room for improvement in avoiding rancid or spoiled food, as only 92.9% of participants reported avoiding such food. Overall, the majority of participants demonstrated correct practices in food safety and hygiene, with 84.4% reporting a good level of practice.

**Table 3:** Practice about food poisoning prevention in food selection and processing (n = 422).

Characteristics		Frequency (n)	Percentage (%)
Buying the food			
***Time to buy food***	Morning	327	77.5
	Afternoon	19	4.5
	Before every meal	72	17.1
	The day before	4	0.9
***Duration to buy food***	Enough	248	58.8
	Temporarily enough	165	39.1
	Lack	9	2.1
**Choosing healthy food**			
Fruits and vegetables should be plump, free from any signs of crushing, with no unusual discoloration, and without any strange or unpleasant odors		405	96
The texture is resilient, firm and smooth, the colors bright		375	88.9
Fresh fish: clear eyes, bright red gills, iridescent scales, firm, resilient texture.		360	85.3
Seafoods are fresh, bright and metallic skin, not fishy		296	70.1
**Eating and drinking cooked food, soak thoroughly, wash vegetables and eat raw**			
Vegetables are soaked in clean water and then washed 3–4 times or washed under running water.		300	71.1
Food is washed and cooked thoroughly.		414	98.1
**Covering and preserving cooked food**			
Eat promptly as soon as prepare foods		390	92.4
Having a table to cover the food		380	90
Having cabinets to store food to avoid flies, cockroaches and insects		385	91.2
There are pesticides and chemicals near the food processing tables		135	32
Thoroughly reheat food to cool before eating		275	65.2
**Separating raw and cooked food, and separating utensils**			
Separate raw and cooked food		290	68.7
Use separate utensils for raw and cooked food		317	75.1
Wash utensils before handling foods		386	91.5
**Sanitary conditions at the food processing tables in the household**			
Kitchen, dining table far from livestock, poultry, toilets		356	84.4
Handling food tables, kitchen without flies, cockroaches, insects		368	87.2
Chemicals, pesticides, pesticides, chemical preparations away from the kitchen		359	85.1
The surface of the handling table is dry and sanitary		391	92.7
Use of water for food processing (Tap water)		397	94.1
The water source for food processing has no color, odor, sediment or strange taste		413	97.9
**Not eating, using rancid, moldy, spoiled food**			
The family avoid food that has signs of rancidity, mold or an unpleasant smell		392	92.9
**Overall practice**			
*Good level of practice*		*356*	***84.4***
*Not good level of practice*		*66*	*15.6*

### 3.4 Multivariable logistic regression model of factors related to knowledge of food poisoning prevention in food selection and processing of study participants

Table 4 shows factors in relation to knowledge of food poisoning prevention in food selection and processing. Household income was found to be associated with knowledge of food caregivers; in detail, the proportion of good knowledge in the high household income group were 3.21 times higher than that in low household income group (OR = 3.21 (1.43–7.16)).

**Table 4:** Multivariable logistic regression model of factors related to knowledge of food poisoning prevention in food selection and processing of study participants.

Factors	Knowledge (*Good*)			Practice (*Correct*)		
	OR	(95% CI)	p	OR	(95% CI)	p
Age group						
≥40	1			1		
<40	1.34	0.78–2.30	0.29	1.48	0.76–2.88	0.24
Occupation						
Housewife	1					
Other	1.38	0.73–2.61	0.32			
Household income classification						
Low	1					
Average	3.21	1.43–7.16	0.004			
Educational level						
Secondary school or less				1		
High school or higher				3.82	1.42–10.31	0.008
Duration of food caregivers						
>10 years				1		
≤10 years				1.30	0.50–3.39	0.58
Knowledge						
*Ingood*				1		
*Good*				5.95	3.35–10.58	<0.001

We found a significant association of education with practice of food poisoning prevention in food selection and processing, that is, food caregivers who attained secondary school or higher were more likely to have a higher proportion of correct practice than that with lower education level (OR = 3.82 (1.42–10.31)). Similarly, food caregivers with good knowledge were more likely to have a higher proportion of correct practice compared to those with poor knowledge (OR = 5.95 (3.35–10.58)).

## 4 Discussion

Along with the socio-economic shift, the global circulation of goods, food safety is one of the issues that need attention because this is an important element of public health. As foodborne illnesses and outbreaks have emerged as a growing concern in recent years, it is important to understand food handlers’ knowledge and practices of food safety. Our study found that 78.9% of the participants showed a good level of knowledge and 84.4% have correct practices related to food poisoning prevention in food selection and preparation in a cross-sectional study with 422 food caregivers. Additionally, positive association of household income with knowledge was observed. Education level and good level of knowledge were identified to have an association with correct practice among food caregivers.

The knowledge and practices in relation to food safety at household levels has been revealed in the existing literature. A previous study documented incorrect safety knowledge and practices among women and emphasized the importance of education programs [8]. The similarity was shared by another study, which reported a deficiency in understanding foodborne illnesses and pathogens; incorrect adherence to proper food hygiene practices, primarily due to mistakes made during both the preparation and storage of food was observed in the majority of households [9]. In contrast, our findings are consistent with another study, which identified a good level of knowledge and practice as well as positive associations between individual knowledge and practice [6]. In comparison with previous studies conducted in central region in Vietnam, our results seem to have a higher level of knowledge and practices [23]. Notably, we place particular emphasis on evaluating food selection and processing methods rather than relying solely on traditional asymptotic approaches to ensure general food safety. In contrast to previous studies that assessed the overall knowledge of food caregivers on a broad range of criteria related to food hygiene and safety, our approach is more targeted and focused. Additionally, our study was carried out in urban areas with favorable economic, cultural, and social conditions, where a strong emphasis has been placed on food hygiene and safety. This has resulted in early implementation of rigorous measures and regular communication campaigns to raise awareness on the topic. It is therefore reasonable to observe a higher level of food safety knowledge and practices in our study compared to previous investigations.

Foodborne illness is a significant public health concern, given its high prevalence and impact worldwide [4]. Ensuring food safety has proven to be a challenge in developing countries due to limited management systems, law enforcement, and knowledge and practices among individuals [18]. Therefore, identifying the factors that influence individuals' knowledge and practices regarding food safety can play a crucial role in reducing the prevalence of foodborne illnesses. Household income was found to have an impact on knowledge of food caregivers in our study. In detail, the rate of good knowledge in the high-income group is 3.21 times higher than in the low-income group (OR = 3.21 (1.43–7.16)). This finding is consistent with previous studies, which documented that individuals with higher income level were more likely to exhibit a higher knowledge of food safety [24,25]. This observation may be explained by the idea that high incomes group is more likely to afford professional training. As a result, they demonstrate higher knowledge levels of food safety [24]. Our finding underscores the significance of taking into account socioeconomic factors when designing and implementing education and awareness programs on food safety. For instance, interventions aimed at enhancing food safety knowledge and practices among low-income food caregivers may require different approaches compared to interventions intended for high-income households.

Additionally, our results also highlight a significant association between education and practice in food selection and processing. Participants with a higher level of education were observed to have a greater food safety practice than those with a lower education level (OR = 3.82 (1.42–10.31)). This is in agreement with a previous study, where education was reported to be an important predictor of health behaviors, including food safety practices [26]. Similarly, another study found that people with low education had lower level of practice compared to the group with a high level of education [27]. This association was also supported by the conclusion from other study [28] Furthermore, knowledge is a factor associated with practice in our study. It suggests the potential role of regular training program to improve knowledge and practices among food caregivers because the trained individuals tend to emerge significantly higher knowledge compared to untrained food handlers [18]. However, it's worth noting that the effectiveness of training programs may be limited to the short-term, as knowledge tends to decrease over time. Thus, we need to put a great emphasis on continuous training to enhance knowledge and practices in the long-term [18].

Our results have some limitations. First, the study used a combination of direct interviews and observation. This made it challenging for interviewers to complete all the assessments, which may have introduced bias. To mitigate this bias, all interviewers were recruited and trained locally to reduce transportation and time management challenges. Second, this study was the first to use a new approach to food safety research. As a result, the standard questionnaire was a limitation, as we adapted all measures from previous studies and the WHO questionnaire. Additionally, we only discussed the overall proportion of knowledge and practice, which adds another limitation due to the many items consisting in each category. Finally, our study was conducted among food caregivers in a specific geographic area, and the results may not be generalizable to other populations.

However, the strength of the study is that it is one of the first to focus on investigating the issue of food selection and processing among food caregivers, who are the primary decision-makers in their families regarding meals preparation. The findings may contribute to providing important insights into the food safety knowledge and practices of food caregivers as initial findings for further research on this topic.

In conclusion, our study provides evidence of high levels of knowledge and practices regarding food safety among food caregivers. The results also highlight the importance of implementing training programs to further improve their knowledge and practices. Furthermore, it is important to consider socioeconomic factors during the design and implementation of such programs to ensure their effectiveness.

## Ethics declarations

This study was reviewed and approved by the Institutional Ethics Council in Biomedical Research of the Hanoi University of Public Health, with the approval number: 13/2022/YTCC-HD3 dated January 17, 2022 (Code: 022-013/DD-YTCC). All participants provided informed consent to participate in the study.

## Author contribution statement

Ngoc Quang La, Binh Thang Tran: Conceived and designed the experiments; Analyzed and interpreted the data; Contributed reagents, materials, analysis tools or data; Wrote the paper.

Minh Luan Hoang, Thi Tao Tran, Cao Khoa Dang: Performed the experiments; Analyzed and interpreted the data; Contributed reagents, materials, analysis tools or data.

## Data availability statement

Data will be made available on request.

## Declaration of competing interest

The authors declare that they have no known competing financial interests or personal relationships that could have appeared to influence the work reported in this paper.
