# Supplementary material for: Assessing the knowledge and practice toward food safety: An investigation of food selection and processing among primary food caregivers in a town of Ha Tinh province, Vietnam
Source: Heliyon. 2023 Sep 9;9(9):e20004. doi: 10.1016/j.heliyon.2023.e20004 (PMC10559741; doi:10.1016/j.heliyon.2023.e20004)
Supplement: Multimedia component 1 [file mmc1.docx]

**Questionnaire on Knowledge and Practice of Food Poisoning Prevention in Selection and Food Preparation of primary food caregivers**

Name:………………………………………………..

Address:……………………………………………………………………………

Date:……………………………………………………………………..

ID:…………………………………………………………….

| 1. **General Information** | |
| --- | --- |
| **A1:** Year of birth | 1. Year……… |
| **A2:** Sex | 1. Male 2. Female |
| **A3:** What is your education level | 1. Illiteracy 2. Elementary school 3. Middle school 4. High school 5. Upper high school |
| **A4:** What is your occupation | 1. Agriculture 2. Small business 3. Government officer 4. Housewife 5. Others |
| **A5:** What is the duration of your homemaker status | 1. <5 years 2. 5-10 years 3. > 10 years |
| **A6:** What is your household income? | 1. Low 2. High |
| **A7:** Where do you hear or receive information about food safety from?  *(Multiple choice)* | 1. Television 2. Speakers/Radio 3. Books, newspapers/magazines 4. Medical staff, women’s union 5. Friends, family members 6. Haven't heard or received information 7. Others: |

| **B1. The knowledge of food poisoning prevention in food selection and processing of housewives**  **B1: 1. Choosing fresh foods** | | |
| --- | --- | --- |
| 1.1. Do you know signs of fresh vegetables? (multiple choice) | | 1.Vegetables are plump, not crushed  2. Reasonable colors  3. Reasonable sense of smell  4. Others  5. Unfamiliar |
| 1.2. Do you know signs of fresh meat?  (multiple choice) | | 1. Bright red color  2. Reasonable sense of smell  3. Resilient  4. Dry slash  5. Others  6. Unfamiliar |
| 1.3. Do you know signs of fresh fish? (multiple choice) | | 1. Clear eyes  2. Bright red gills  3. Bright red slash  4. Iridescent scales  5. Others  6. Unfamiliar |
| **2. Eating cooked drinking boiled, soaking, and washing raw vegetables and fruits** | | |
| 2.1. Vegetables should be soaked in clean water and then washed 3-4 times or washed under running water | | 1. Yes 2. No 3. Unfamiliar |
| 2.2. Food should be cooked thoroughly? | | 1. Yes 2. No 3. Unfamiliar |
| **3. Eat meal immediately after cooking or right after it has been prepared** | | |
| 3.1. When food is cooked*,* it should be eaten promptly? | | 1. Yes 2. No 3. Unfamiliar |
| 3.2. Do you know leaving cooked food to cool for a long time is susceptible to food poisoning? | | 1. Yes 2. No 3. Unfamiliar |
| 3.3. Do you know that nutrient-rich dishes such as meat, fish, eggs, etc., after being processed, which can cause food poisoning if not eaten immediately and well preserved? | | 1. Yes 2. No 3. Unfamiliar |
| **4. Covering up and carefully preserving food after cooking** | | |
| 4.1. Do you know that food should be covered to avoid flies, and, cockroaches, rats? | 1. Yes 2. No 3. Unfamiliar | |
| 4.2. Do you know that leaving fertilizers, pesticides… near food can cause poisoning? | 1. Yes  2.No  3. Unfamiliar | |

| **5. Carefully reheating leftovers before using again** | |
| --- | --- |
| 5.1. Do you know that it is hygienic to reheat food before eating? | 1. Yes 2. No 3. Unfamiliar |
| 5.2. Do you know that food can be contaminated when it is cool? | 1. Yes 2. No 3. Unfamiliar |
| **6. Separating raw and cooked food, and separating utensils to prepare food** | |
| 6.1. Do you know that raw foods are often contaminated with disease-causing microbes? | 1. Yes 2. No 3. Unfamiliar |
| 6.2. Do you know that cross-contamination occurs when juices from uncooked foods come in contact with safely cooked foods | 1. Yes 2. No 3. Unfamiliar |
| 6.3. Do you know that use different utensils, plates and chopping boards for raw and cooked food | 1. Yes 2. No 3. Unfamiliar |
| 6.4. Do you know that wash utensils before preparing food? | 1. Yes 2. No 3. Unfamiliar |
| 6.5. Do you know that leftover should be stored covered and remove daily? | 1. Yes 2. No 3. Unfamiliar |
| **7. Washing hands before handling food, keeping utensils and food preparation tables dry** | |
| 7.1. Do you know that washing hands before handling foods and after using the restroom? | 1. Yes 2. No 3. Unfamiliar |
| 1. **Keep utensils and the food preparation area dry** | |
| 8.1. Do you know that food preparation tables must be dry, far from latrines, chicken, duck and pig coops at least > 10m? | 1. Yes 2. No 3. Unfamiliar |
| 8.2. Do you know that places of eating, processing, pantry with flies, cockroaches and insects are unhygienic? | 1. Yes 2. No 3. Unfamiliar |
| 8.3. Do you know that keep food preparation tables away from disinfectants, disinfectants, fertilizers, pesticides? | 1. Yes 2. No 3. Unfamiliar |
| 8.4. Do you know that food preparation tables need maintained dry and sanitary? | 1. Yes 2. No 3. Unfamiliar |
| **9. Do not eat or use food that is spoiled, moldy, or rotten** | |
| 9.1. Do you know Using spoiled food can cause illness? | - 1. Yes   2. No   3. Unfamiliar |
| 9.2. Do you know Leaving cooked food to cool for a long time is susceptible to food poisoning? | 1. Yes 2. No 3. Unfamiliar |
| **10. Prepare food using clean water** | |
| 10.1 Do you know that lack of clean water for processing food will be unhygienic | 1. Yes 2. No 3. Unfamiliar |

Thank you for your cooperation **Investigator**

(signature)

**Part II: Practice about food poisoning prevention in food selection and processing (observation)**

**Buying the foods**

1. Time to buy foods?

1. Morning 2. Afternoon

3. Before every meal 4. The day before

**2**. How do you evaluate the time spent on carefully selecting food when you go grocery shopping?

1. Enough 2. Temporarily enough 3. Lack

**Choosing healthy food**

1. Fruits and vegetables should be plump, free from any signs of crushing, with no unusual discoloration, and without any strange or unpleasant odors?

1. Yes 2. No

1. The texture is resilient, firm and smooth, the colors bright?
2. Yes 2. No
3. Fresh fish: clear eyes, bright red gills, iridescent scales, firm, resilient texture.
4. Yes 2. No
5. Seafoods are fresh, bright and metallic skin, not fishy?
6. Yes 2. No

**Eating and drinking cooked food, soak thoroughly, wash vegetables and eat raw**

1. Vegetables are soaked in clean water and then washed 3-4 times or washed under running water.
2. Yes 2. No 3. Others
3. Food is washed and cooked thoroughly?

1. Yes 2. No 3. Others

**Covering and preserving cooked food**

1. Eat promptly as soon as prepare foods?

1. Yes 2. No

**Cover and store cooked food**

1. Check if there is a food cover/cloche available?

1. Yes 2. No

1. Check if there are cabinets to store food to avoid flies, cockroaches and insects?

1. Yes 2. No

1. Check if there are pesticides and chemicals near the food processing tables

1. Yes 2. No

**Reheat food thoroughly before consumption**

1. Check if foods have been left to cool be thoroughly reheated before eating?

1. Yes 2. No

**Do not mix raw and cooked foods, and do not use the same utensils for food preparation**

1. Is raw food mixed with cooked food?

1. Yes 2. No

1. Do you use the same utensils for preparing raw and cooked food?

1. Yes 2. No

1. Do you clean the utensils before using them for food preparation?

1. Yes 2. No

**Wash hands before cooking and before eating**

1. Do you wash your hands before directly handling cooked food?

1. Yes 2. No

**Place for utensils, food preparation area**

1. Is the kitchen, dining table close to livestock pens, poultry, restrooms, etc.?

1. Yes 2. No

1. Are there flies, cockroaches, or insects in the food preparation area, kitchen?

1. Yes 2. No

1. Are chemicals, pesticides, plant protection products, or other chemical substances kept near the kitchen?

1. Yes 2. No

**21. Are the surfaces in the food preparation area clean and dry?**

1. Yes 2. No

**Do not eat or use food that is spoiled, moldy, or rotten**

**22**. Does the family use food that shows signs of spoilage, mold, or has an unpleasant odor?

1. Yes 2. No

**Prepare food using clean water**

1. What water source does the family primarily use for food preparation

1. Tap water 2. Well water 3. River water

4. Rainwater 5. Pond water 6. Others

1. Is the water discolored, has an unusual smell, sediment, or strange taste?

1. Yes 2. No
